# A Dual-Plasmid-Based CRISPR/Cas9-Mediated Strategy Enables Targeted Editing of pH Regulatory Gene *pacC* in a Clinical Isolate of *Trichophyton rubrum*

**DOI:** 10.3390/jof8121241

**Published:** 2022-11-24

**Authors:** Sanchita Sanchaya Dey, Sivaprakash Ramalingam, Bhupesh Taneja

**Affiliations:** 1 CSIR-Institute of Genomics and Integrative Biology (CSIR-IGIB), New Delhi 110025, India; 2 Academy of Scientific and Innovative Research (AcSIR), Ghaziabad 201002, India

**Keywords:** dermatophytes, CRISPR/Cas9, NHEJ repair, *Trichophyton rubrum*, genome editing, *pacC*

## Abstract

*Trichophyton rubrum* is the most prevalent causative agent responsible for 80–90% of all known superficial fungal infections in humans, worldwide. Limited available methods for genetic manipulations have been one of the major bottlenecks in understanding relevant molecular mechanisms of disease pathogenesis in *T. rubrum*. Here, a dual-plasmid-based CRISPR/Cas9 strategy to edit pH regulatory transcription factor, *pacC*, of a clinical isolate of *T. rubrum* by non-homologous end joining (NHEJ) repair is presented. A cas9–eGFP fusion that aids pre-screening of primary transformants through detection of GFP fluorescence is expressed from one plasmid while target-specific sgRNA from the other brings about mutagenesis of *pacC* with an overall efficiency of 33.8–37.3%. The mutants had reduced transcript levels of *pacC* at both acidic and alkaline pH with several morphological abnormalities. We believe this dual-plasmid-based CRISPR/Cas9 strategy will aid functional genomics studies, especially in non-lab-adapted clinical strains of *T. rubrum*.

## 1. Introduction

Dermatophytes are a highly specialized group of keratinophilic fungi which invade the stratum corneum causing chronic skin, hair and nail infections [1,2]. These pathogenic fungi cause superficial skin infections on different parts of body. Among dermatophytes, *T. rubrum* is the leading cause of infections on skin and nails in humans, and is alone responsible for 80–90% of all known superficial fungal infections [3,4,5,6,7]. In recent years, increased infectivity, poor therapeutic response, and high recurrence rate of these pathogenic fungi has necessitated the need to adapt modern and new reverse genetic approaches to understand the underlying mechanisms of emerging drug resistance and key gene functions [8,9,10,11,12].

Elucidation of the molecular basis of pathogenesis, invasion, and treatment in dermatophytes has largely remained obscure because of limited availability of efficient tools and difficulty performing large-scale gene knockouts in non-model albeit clinically important filamentous fungi, viz., *T. rubrum* [13,14,15]. Moreover, the low frequency of homologous recombination in dermatophytes had prevented earlier attempts at generation of mutants in *Microsporum canis* and *Trichophyton mentagrophytes* [16].

Although successfully generated gene knockouts of *T. rubrum* are scarce, a few reports of gene knockout in other closely related dermatophytes, by homology-directed repair (HDR) or gene silencing by RNA interference are available. For instance, targeted gene disruption of *dnr1*, the *areA/nit-2*-like nitrogen regulatory gene of the zoophilic dermatophyte *M. canis* could be achieved, although only 2/100 transformants were successfully mutated [17]. Inactivation of ABC transporter *TruMDR2* in *T. rubrum* [18] and the pH-associated transcription factor *pacC* in *T. rubrum* [19] are among the other early available gene knockout reports from the anthropophilic dermatophyte. An alternative approach to study gene function, using RNA silencing, was demonstrated for SUB3 and DPPIV in *M. canis* that suppressed the expression of the secreted proteases from 5% to 100% among different transformants [20]. Although alternate approaches, such as the *Agrobacterium tumefaciens*-mediated transformation system, were also used to overcome low transformation frequencies and facilitate DNA transfer to generate genetic mutants of the *areA/nit-2*-like regulatory gene (*tnr*) in *T. mentagrophytes*, low frequency of homologous recombination (0–30%) continued to be reported [21]. To overcome this issue, non-homologous end-joining (NHEJ)-deficient *ku80* mutants of *T. mentagrophytes* [8,21] were found to increase the efficiency of mutants from 2.2% in the parent strain to nearly 70% in the *ku80^-^ T. mentagrophytes* strain [8]. However, one cannot rule out the risk of unforeseen genetic variations in the mutants deficient in NHEJ machinery [14]. Moreover, the prerequisite of a genetically modified strain to obtain a higher frequency of homologous recombination to generate gene knockouts has limited the functional characterization to only a small subset of genes among dermatophytes thus far. Hence, a need to find an alternative and more efficient tool for target-specific gene modifications in *T. rubrum* continues to exist for these clinically important filamentous fungi.

Targeted gene manipulation with the help of a single guide RNA (sgRNA)-guided Cas9 nuclease has made CRISPR/Cas9 a powerful gene editing tool in several cellular systems. Recently, a Cas9–sgRNA ribonucleoprotein (RNP) complex-mediated gene disruption of *ura3* in *T. rubrum* was reported that enables the extrapolation of auxotrophic mutants for genetic studies from model fungal species (viz., *S. cerevisiae* and *C. albicans*) to non-model filamentous fungi [22] and shows that CRISPR/Cas9 methods can be adapted for gene manipulations in this pathogen. 

Dermatophytes thrive in a wide range of pH variations in their immediate microenvironment at the infection site. Upon infection by its conidia, they release various adhesins, keratinases, proteases, and enzymes which are active at acidic skin pH, and thus aid the breakdown of keratins and other proteins to avail nutrition [23]. This process leads to the release of ammonia as a by-product of amino acid metabolism, thereby shifting extracellular pH from acidic to alkaline [24]. PacC enables the adaptation to this shift in pH and gene disruption of *pacC* earlier exhibited reduced keratinase secretion, decreased growth on human nails, and abnormal conidiation that could be monitored in vitro [19].

In the present work, a dual-plasmid-based CRISPR/Cas9 strategy that uses a Cas9 and enhanced-Green Fluorescent protein (eGFP) fusion (Cas9GFP) [25] to edit *pacC* of a clinical strain *T. rubrum* IGIB-SBL-CI1 is reported. The Cas9GFP aids pre-screening of primary transformants through detection of GFP fluorescence while the positive transformants were monitored for their adaption to shift in pH after the confirmation of mutations using a T7 endonuclease assay and sequencing. Confirmed *pacC* mutants exhibited decreased growth, altered conidiation abilities, hyperpigmentation, and reduced *pacC* expression. The dual-plasmid-based strategy described here has the potential for direct application of gene editing in non-lab-adapted clinical strains and is expected to find wide use for establishing key gene functions in *T. rubrum* and other non-model filamentous fungi.

## 2. Methods

### 2.1. Strains, Plasmids, and Growth Conditions

A previously isolated clinical strain available in the lab, *T. rubrum* IGIB-SBL-CI1, was used in this study as the parent strain [26]. The strain has been maintained with less than three passages to prevent lab adaptations. The morphology, phenotype, and microscopic examination of *T. rubrum* IGIB-SBL-CI1 was carried out using 10% KOH as well as on a lactophenol cotton blue mount and found to be typical of *T. rubrum* strains [27,28,29]. The genotypic identification of *T. rubrum* IGIB-SBL-CI1 was confirmed by sequencing the internal transcribed spacer regions 1–2 of 18S rRNA (NCBI Accession ID: OL614127). This isolate harbors a c.1179A>T mutation corresponding to a p.L393F amino acid substitution in *erg1* (NCBI Nucleotide ID: HL42_2512, NCBI protein_id: KMQ46827.1) (Table S1) when compared to *erg1* (NCBI Nucleotide ID: TERG_05717) of Refseq strain, *T. rubrum* CBS 118892), and exhibits resistance to terbinafine (BT, personal communication). The plasmid psgRNA5.0 [30] was used to clone target-specific sgRNA; pCas9GFP was used for in vivo Cas9 expression while pT7-sgRNA [31] was used for in vitro cleavage assays. The Cas9GFP in pCas9GFP is codon-optimized for expression in *Aspergillus* spp. and hence other closely related fungi belonging to *Ascomycetes* (viz., dermatophytes) [25]. The green fluorescence of eGFP-tagged Cas9 (Cas9GFP) was used as an aid for pre-screening of transformants [25]. All plasmids were maintained in *Escherichia coli* DH5α in Luria–Bertani broth containing ampicillin (100 μg/mL) at 37 °C. The strains, plasmids, oligonucleotides, and primers used in the study are listed in Table S1.

*T. rubrum* IGIB-SBL-CI1 was cultivated on Sabouraud’s dextrose broth (SDB) and Sabouraud’s dextrose agar (SDA) plates at 30 °C for 10 days or on minimal media (MM) for 3–4 weeks. The complete composition of MM used was as defined by [32]. The selection of fungal transformants was carried out on SDA or MM agar plates supplemented with 10 mM acetamide and 15 mM cesium chloride (for amdS selection) as defined by [25] along with 100 µg/mL geneticin (for *neo*^+^ selection) and hence designated as SDA selection media (SSM) or minimal media selection agar (MMSA), respectively. The selected concentration of geneticin completely inhibited the growth of the parent *T. rubrum* IGIB-SBL-CI1 strain.

### 2.2. sgRNA Plasmid Construction

*pacC* in *T. rubrum* IGIB-SBL-CI1 (NCBI Nucleotide ID: HL42_7193) was identified with 100% nucleotide identity corresponding to *pacC* of the Refseq strain, *T. rubrum* CBS118892 (NCBI Nucleotide ID: XM_003238803.1), consisting of 3 exons and 2 introns that resulted in a gene product of 815 residues (NCBI Protein IDs: XP_003238851.1 for *T. rubrum* CBS 118892 and KMQ42134.1 for *T. rubrum* IGIB-SBL-CI1). The target sites for the recruitment of Cas9 nuclease were selected manually on the basis of the following features described earlier [33], namely, (i) the presence of a unique 20 bp long potential cleavage site upstream of a 5’-NGG-3’ protospacer adjacent motif (PAM), (ii) the selection of the target site near the 5′ end of the coding DNA sequence (cds) for higher likelihood of early frame shifts and the generation of stop codons in the edited gene product, and (iii) the absence of any predicted off-target sites in the genome. Four sets of oligonucleotides, sgRNA-1, sgRNA-2, sgRNA-3, and sgRNA-4, with the above features, were hence designed to target exon 1 and exon 2 of *pacC* as indicated (Figure S1). For the cloning of sgRNA-1-4, each pair of sgRNA oligonucleotides was synthesized with BbsI overhangs (5′-CACC-3′, forward primers, and 5′-AAAC-3′, reverse primers), annealed at 55 °C for 30 min and ligated into BbsI-digested psgRNA5.0 (to yield psgRNA5_sg1, psgRNA5_sg2, psgRNA5_sg3 and psgRNA5_sg4) or into pT7sgRNA (to yield pT7sgRNA_sg1, pT7sgRNA_sg2, pT7sgRNA_sg3 and pT7sgRNA_sg4), as described earlier [30]. The respective constructs were transformed into, and purified from, *E. coli* DH5α to confirm the clones using Sanger sequencing.

### 2.3. DNA Cleavage by In Vitro Assembled Cas9–RNP Complex

To check the specificity of sgRNA to recognize the target region, an in vitro cleavage assay was performed. The respective pT7sgRNA constructs (pT7sgRNA_sg1, pT7sgRNA_sg2, pT7sgRNA_sg3 and pT7sgRNA_sg4) were first linearized by BamH1 before carrying out in vitro transcription (IVT) using the Megascript T7 transcription kit ( ThermoFisher, Waltham, MA, USA). The in vitro transcribed guide RNA products (IVTsgRNA-2, IVTsgRNA-3, and IVTsgRNA-4 from pT7sgRNA_sg2, pT7sgRNA_sg3, and pT7sgRNA_sg4, respectively) of an approximate size of 100–150 bp (20 bp sgRNA + ~100 bp RNA scaffold) were obtained and purified using Nucaway spin columns. pT7sgRNA_sg1 did not yield a product and was not considered for further experiments. The in vitro transcribed guide RNA products were heated at 92 °C for 2 min to separate the two strands and incubated with an equimolar concentration (250 nM each) of *S. pyogenes* Cas9 nuclease (spCas9) [34] at 25 °C for 10 min to constitute Cas9–sgRNA RNP complexes in 1 × Protein reaction buffer (1 M NaCl, 0.1 M MgCl_2_, 0.5 M Tris-HCl, pH 7.9, and 1 mg/mL BSA).

To carry out the in vitro cleavage assay, DNA substrates were obtained using PCR amplification of different regions of *pacC* encompassing the target sites for sgRNA1-4, namely, G1 [−319 to +286], G2 [−319 to +408], G3 [+267 to +945], and G4 [+389 to +1016] (Table S2). A total of 5nM of PCR products of G1, G2, G3, and G4 were incubated with the respective 250 nM RNP reaction mix at 37 °C for 30 min for the cleavage. The reaction was terminated by adding 2 μL of 10mg/mL Proteinase K at 55 °C for 20 min and the cleavage products checked on a 1% agarose gel using gel electrophoresis.

### 2.4. Protoplast Generation, Transformation of T. rubrum, and Mutant Screening

Protoplasts of *T. rubrum* IGIB-SBL-CI1 were generated as described previously with minor modifications [35]. First, conidia were isolated by gently scraping the surface of the fungal colony with a sterile spatula. The harvested mycelia along with conidia was suspended in 5 mL sterile distilled water and filtered through a double layered muslin cloth to remove the hyphal fragments. The purified conidia were then counted using a hemocytometer and 10^7^ conidia were inoculated in 10 mL SDB and incubated at 30 °C for 5 days on an orbital shaker at 200 rpm to induce germination. Germinated conidia were next harvested and filtered through a sterile muslin cloth after confluent growth and incubated with 10 mg/mL Yatalase (Takara Bio, Japan) for cell wall digestion in digestion buffer (0.6 M MgSO_4_, 25 mM CaCl_2_, and 50 mM malate buffer, pH 5.5) at 30 °C for 6 h. The resulting protoplasts were filtered through sterile muslin cloth to separate the mycelial debris and pelleted by centrifugation at 2000× *g* for 15 min at 4 °C. Protoplasts were washed with STC buffer (1.2 M sorbitol; 10 mM Tris-HCl, pH 8.0; 10 mM CaCl2) twice and finally resuspended in STC buffer at a concentration of 10^7^ protoplasts/mL. The protoplast suspension was stored at 4 °C until further use.

For the transformation reaction, 100 μL protoplast suspension was incubated with 5 μg of psgRNA5.0 encoding the respective sgRNA along with 5 μg pCas9GFP on ice for 10 min. In order to aid protoplast fusion and DNA uptake, 1200 µL of polyethylene glycol (PEG) buffer (25% PEG 3350, 20 mM CaCl_2_, and 10 mM Tris–HCl, pH 8.0) was added in incremental steps to the transformation reaction mix and incubated on ice for an additional 10 min. The transformation reaction mix was then centrifuged at 3000× *g* in a swinging bucket centrifuge and washed again with STC buffer thoroughly to prevent any cytotoxicity from remnants of PEG. The transformation reaction mix was then resuspended in 400 μL of STC buffer, plated onto SSM plates, and incubated at 30 °C for 10 days. The resulting transformants were subcultured on MMSA plates for two generations to ensure mitotic stability. Finally, transformants were screened by monitoring GFP expression using a Leica SP8 laser scanning confocal microscope with the excitation wavelength set to 488 nm and emission measured in the range of 500–550 nm. 

Next, a T7 endonuclease (T7E1) assay [36] of GFP-expressing transformants was carried out for the detection of mutants. Furthermore, G2, G3, and G4 amplicons of confirmed transformants were sent for Sanger sequencing and the chromatograms analyzed by two independent decomposition algorithms TIDE (https://tide.nki.nl/, accessed on 29 July 2021) [37] and ICE (https://ice.synthego.com/#/, accessed on 13 August 2021) [38] to assess the NHEJ mutants and to determine mutation frequencies. Trace files were analyzed with a *p* < 0.0001 cut-off value and output files with an R^2^ < 0.90 were eliminated.

### 2.5. Off-Target Analysis

In order to identify potential off-target sites, any off-target binding of single-guide RNAs (sgRNA-3 and sgRNA-4) in the genome of *T. rubrum* were searched with several widely used genome editing and off-target identification tools. CHOPCHOP (https://chopchop.cbu.uib.no/, accessed on 26 May 2022) predicted that there are no off-targets for sgRNA-3 and identified only one potential off-target site for sgRNA-4 [39]. Additional potential off-target sites were shortlisted using a lower stringency, i.e., 70% or more sequence identity (using BLASTN with a penalty score of −1) for sgRNA-3 and sgRNA-4. Four potential off-target sites were finally predicted which were analyzed using PCR amplification followed by Sanger sequencing.

### 2.6. Quantitative Reverse Transcriptase Polymerase Chain Reaction (qRT-PCR)

To measure the change in expression levels of *pacC*, as a function of pH variation, transcript levels of *pacC* in respective mutants of *T. rubrum* IGIB-SBL-CI1 were measured using qRT-PCR. First, the parent *T. rubrum* IGIB-SBL-CI1 strain and *pacC* mutants were grown in SDB for 72 h and subjected to pH shift by transferring to SDB buffered with sodium-citrate, pH 4.0, or Tris-HCl, pH 8.0, for 3 h. cDNA was then synthesized with Superscript IV reverse transcriptase (Invitrogen, Carlsbad, CA, USA) using random hexamer primers, following the manufacturer’s instructions. The qRT-PCR was finally performed using SYBR Premix Ex Taq (Takara Bio, Kusatsu, Japan) with *β-actin* as an internal control. The expression of each gene was measured in three technical replicates and for each sample three biological replicates were set up using specific primers (Table S1). The fold change in gene expression levels was calculated using the 2^−ΔΔCT^ method, as described earlier [40].

### 2.7. Phenotypic Characterization of pacC Mutants

SDA as well as MM-agar plates were point inoculated with 1 × 10^3^ spores of parent *T. rubrum* IGIB-SBL-CI1 strain or *pacC* mutant strains and incubated at 30 °C for 14 days. The growth was monitored by measuring the diameter of mycelial extension (mm) for three independent replicates. The growth was similarly monitored as a function of pH variation (i.e., SDA buffered at pH 4.0 or pH 8.0) and as a function of saline stress (i.e., SDA containing 0.5 or 0.8 M NaCl). To monitor conidiation, conidia were isolated from 14-day-old cultures grown on SDA and counted under a microscope with a hemocytometer for three independent replicates.

To study the pigmentation pattern, parent and mutant strains were inoculated with 1 × 10^3^ spores in SDB (with shaking at 200 rpm) at 30 °C for 14 days and examined visually.

## 3. Results

### 3.1. Overall Strategy

**Figure 1:**
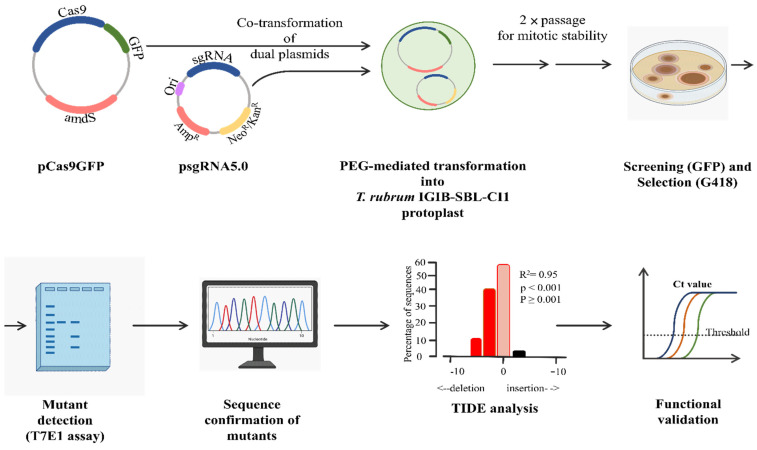
Schematic illustration of dual-plasmid-based CRISPR/Cas9 strategy for gene knockout of pacC. sgRNAs were designed and cloned in psgRNA5.0. Co-transformation by PEG-mediated method, with pCas9gfp, yields primary transformants. GFP-expressing mitotically stable transformants were screened on selection media. Cas9-GFP generates a double stranded break guided by pacC-specific sgRNAs. An error prone NHEJ repair results in mutants. Mutant detection by T7E1 was followed by further confirmation by sequencing and validation of loss of function of pacC in vivo.

In order to generate an NHEJ-mediated gene knockout in *T. rubrum* using the CRISPR/Cas9 approach, two different plasmids, pCas9GFP (to express the eGFP-tagged cas9 nuclease, Cas9GFP) and psgRNA5.0 (to express gene-specific sgRNAs), were used to target *pacC* of *T. rubrum* IGIB-SBL-CI1 (Figure 1). Four different sgRNAs were designed; sgRNA-1 and sgRNA-2 complementary to exon1 and sgRNA-3 and sgRNA-4 complementary to exon2 of *pacC* (Figure S1). In vivo expressed sgRNA would form a ribonucleoprotein complex with Cas9GFP and identify the target sequence next to a PAM site, with high specificity (Figure S1). The sgRNA–Cas9GFP RNP complex would next generate a double-stranded break three bases upstream of PAM, resulting in mutants through an error-prone NHEJ repair at the break site. The resulting mutants were screened on selection media and confirmed using the T7 endonuclease (T7E1) assay followed by gene sequencing and validation. Subsequently, the mutant strains were subjected to expression analysis of *pacC* followed by phenotypic and morphological examination of the mutant colonies. The overall dual-plasmid-based strategy for targeted gene editing of *pacC* in *T. rubrum* IGIB-SBL-CI1 is illustrated in Figure 1.

### 3.2. In Vitro Cleavage Assay (IVC)

**Figure 2:**
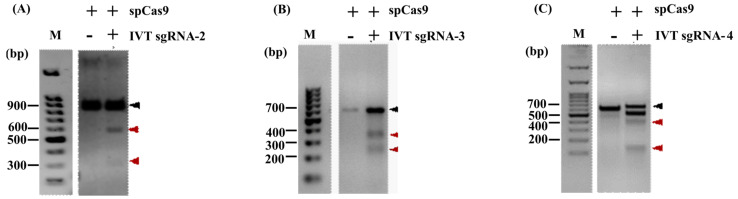
Cleavage assay. An in vitro cleavage assay with in vitro transcribed guide RNA products and spCas9 was carried out. (**A**) IVTsgRNA-2, (**B**) IVTsgRNA-3, and (**C**) IVTsgRNA-4. The respective amplicons were cleaved by spCas9 to yield bands of expected sizes, while sgRNA-1 did not express (not shown). The uncleaved DNA and cleaved fragments are indicated by black and red arrows, respectively. A 100 bp DNA ladder was run separately for each cleavage reaction and is shown for reference. Sizes of uncut and cleaved products are given in Table S2.

In order to check the specificity and efficiency of designed sgRNAs to recognize the target region, in vitro transcribed guide RNA products, IVTsgRNA-2, IVTsgRNA-3, and IVTsgRNA-4, were incubated with G2, G3, and G4 regions of *pacC* (Table S2) as substrates for an in vitro cleavage assay with spCas9, as described in the Methods section. All three in vitro transcribed guide RNA products resulted in cleaved DNA fragments of expected size (Figure 2) indicating that all three designed sgRNAs are capable of efficient cleavage at specific target sites in vitro. The in vitro cleavage assay, hence, provided a successful pre-validation for further use of sgRNA-2, -3, and -4 for gene disruption of *pacC* in vivo.

### 3.3. Dual-Plasmid-Based CRISPR/Cas9 Strategy Successfully Generates pacC Mutants of T. rubrum IGIB-SBL-CI1

**Figure 3:**
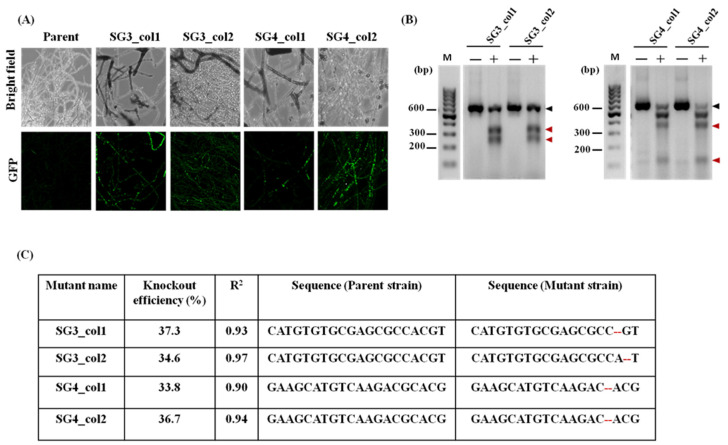
Generation of pacC mutants of T. rubrum IGIB-SBL-CI1. (**A**) GFP fluorescence for two randomly selected mitotically stable transformants obtained with sgRNA-3 (SG3_col1, SG3_col2) and sgRNA-4 (SG4_col1, SG4_col2). BF indicates brightfield while GFP indicates fluorescence measured in the range of 500–550 nm. (**B**) T7 endonuclease (T7E1) assay for transformants SG3_col1 and SG3_col2 (left panel) as well as SG4_col1 and SG4_col2 (right panel) confirms targeted NHEJ repair in pacC in all four transformants. Uncleaved DNA is indicated by black arrows and the cleaved products by red arrows. Sizes of uncut and cleaved products are given in Table S2. (**C**) The frequency of mutations along with indels, as identified by TIDE and ICE analysis tools, are indicated. The goodness of fit for the sequence trace decomposition, R^2^, as estimated by TIDE [37] is also indicated.

In order to carry out targeted gene editing of *pacC*, protoplasts of *T. rubrum* IGIB-SBL-CI1 were co-transformed with pCas9GFP and with psgRNA5_sg2, psgRNA5_sg3, or psgRNA5_sg4 (harboring the corresponding pre-selected sgRNA-2, sgRNA-3, or sgRNA-4, respectively) in three different reactions. Primary transformants (approximately 200–250 for each co-transformation reaction) were obtained on SSM plates; 20 of these were subcultured on MMSA plates for two generations to ensure mitotic stability before further screening. A total of 16/20 (80%) mitotically stable colonies for pCas9GFP-psgRNA5_sg2, 16/20 (80%) for pCas9GFP-psgRNA5_sg3, and 13/20 (65%) for pCas9GFP-psgRNA5_sg4 were thus obtained. Mitotically stable clones were randomly screened for transient expression of GFP. Although no GFP fluorescence was obtained for transformants resulting from the pCas9GFP-psgRNA5_sg2 co-transformation reaction, transformants resulting from pCas9GFP-psgRNA5_sg3 and pCas9GFP-psgRNA5_sg4 exhibited GFP fluorescence (Figure 3A). As psgRNA5_sg3 and pCas9GFP-psgRNA5_sg4 indicated positive transformation reactions, the colonies were confirmed for Cas9-mediated mutations among these transformants by the T7E1 assay. The T7E1 assay with target regions G3 and G4 yielded cleavage products of expected sizes in 5/16 colonies for sgRNA-3 and 4/13 colonies for sgRNA-4, confirming the NHEJ-mediated repair at the target sites (Figure 3B). 

Mutations in the target regions were confirmed using Sanger sequencing and indicated that Cas9-mediated mutations in *pacC* were successfully generated at the target site in all the screened colonies. The efficiency of mutations was estimated by analyzing the sequencing chromatograms with TIDE webtool. Indels were also estimated using ICE server and were found to be congruent. All *pacC* mutants contain a 2bp deletion 1-3 bp upstream of PAM region while no large indels were identified in any of the mutants. The total efficiency of mutants varied from 33.8% to 37.3% (Figure 3C, Figure S2) with a high goodness of fit (R^2^ > 0.90). As all colonies exhibited similar 2 bp deletions (Figure 3C, Table S3), two colonies each, annotated as SG3_col1, SG3_col2, SG4_col1, and SG4_col2, were selected for further characterization. The 2bp deletion leads to a frameshift, resulting in truncated protein products of 199 residues from SG3_col1 or SG3_col2 and 250 residues from SG4_col1 or SG4_col2 mutants in contrast to 815 residues for full-length PacC. PCR amplification followed by Sanger sequencing of predicted off-target sites did not reveal any off-target mutations (Figure S3) in these genomes. These results suggest that the CRISPR/Cas9 system used here would be efficient to induce targeted mutagenesis of other genes in *T. rubrum* and in related dermatophytes as well.

### 3.4. Characterization of the pacC Mutants

The effect of *pacC* mutation was monitored using direct macromorphological observations of colony variations (i.e., radial growth measurements, conidiation, and melanin production), and expression analysis of *pacC*, in the respective mutant strains. 

**Figure 4:**
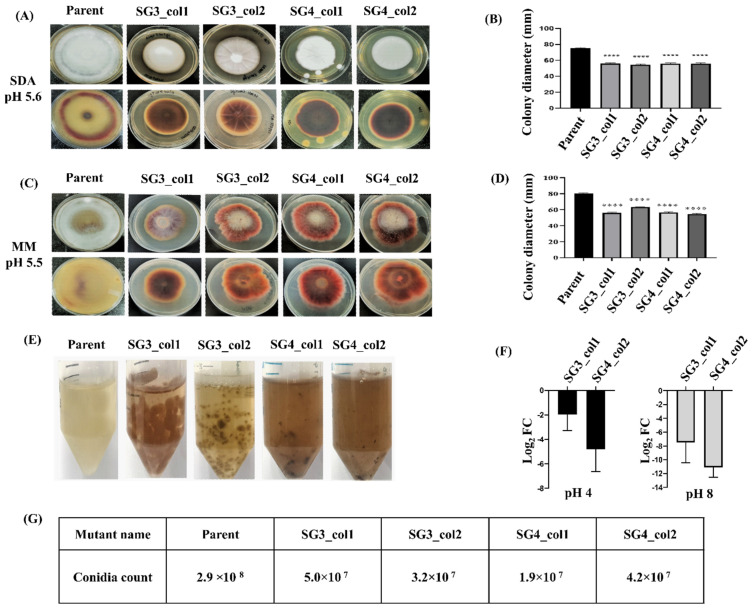
Phenotypic characteristics and *pacC* expression in mutant strains. Growth of *pacC* mutants and the parent *T. rubrum* IGIB-SBL-CI1 strain was monitored on (**A**) SDA plates (pH 5.6) and (**C**) MM agar plates (pH 5.5). The top and bottom panels in both (**A**) and (**C**) are images of front and reverse sides of the agar plates, respectively. Average colony diameters (in mm) measured from three different plates of each mutant are plotted for growth on (**B**) SDA plates and (**D**) MM agar plates along with one-way ANOVA with Tukey’s post-hoc test (**** *p* < 0.0001). (**E**) Hyperpigmentation, as seen in mutant strains in SDB. (**F**) Gene expression profiling represented as log_2_-fold change in *pacC* expression in mutant strains using *T. rubrum* IGIB-SBL-CI1 as a reference, at alkaline and acidic pH conditions. The average values of three independent experiments are plotted after normalization with the *β-actin* endogenous gene; error bars indicate ± standard deviation. (**G**) Conidia count of the four *pacC* mutants is nearly 10-fold less than the parent strain, on SDA plates.

The mean diameter of all the four mutant strains with a confirmed 2 bp deletion, i.e., SG3_col1, SG3_col2, SG4_col1, and SG4_col2 was somewhat smaller (55.4 ± 0.7 mm for all four mutants) than the mean diameter of the parent strain (75.3 ± 0.6 mm) after 14 days of growth on nutrient-rich media (SDA) (Figure 4A, B). The radial growth of all four mutant strains was also monitored on MM agar plates after 21 days of growth (Figure 4C) and showed a trend similar to when grown on SDA plates, i.e., reduced growth with a smaller mean diameter (57.6 ± 3.9 mm for all four mutants) as compared to the parent strain (80 ± 1 mm) (Figure 4D). While the mycelia of all four *pacC* mutants showed the usual white-cottony appearance as seen in the parent strain on SDA plates, the characteristic white-cottony mat of *T. rubrum* strains was completely absent in the mutants on MM agar plates (Figure 4C). 

The mutant strains also produced evident alterations on the mycelium pigmentation on both SDA and MM agar plates. The mutants SG3_col1, SG3_col2, SG4_col1, and SG4_col2 developed a reddish-brown pigmentation on nutrient-rich SDA and SDB or on MM agar plates in contrast to a light yellow to yellowish-brown pigmentation seen in the parent strain (Figure 4A,C,E), indicating an overall alteration in characteristic pigmentation among *pacC* mutant strains. 

As the mutant strains, SG3_col1, SG3_col2, SG4_col1, and SG4_col2 exhibited similar phenotypic characteristics; SG3_col1 and SG4_col2 were selected to measure the change in expression levels due to *pacC* mutation. The transcript levels of *pacC* in SG3_col1 and SG4_col2 were evaluated using qRT-PCR upon shift to pH 4.0 or pH 8.0. The qRT-PCR analysis indicated low *pacC* transcripts in both the mutants during growth at either pH (Figure S4), suggesting that small amounts of (possibly unstable) mRNA transcripts produced in the mutant strains could be detected in these strains as the primer designed to monitor *pacC* expression corresponds to exon1 (Table S1) and would be able to bind to any truncated RNA produced in the mutant strains. log_2_ transformed fold changes upon shift to acidic pH ranged from −4 to −1.9 in SG3_col1 and SG4_col2, respectively, indicating a marked reduction in transcript levels. (Figure 4F). At alkaline pH, however, the change in expression levels of *pacC* is even more discernible with a log_2_fold transformed fold change ranging from −11 in SG3_col1 to −9 in SG4_col2, correlating with the higher expression levels of pacC at pH 8.0 in the parent strain (Figure 4F, Figure S4), possibly due to the role of pacC in modulating the growth of *T. rubrum* at alkaline pH. 

Mycelia of all four *pacC* mutants showed the usual white-cottony appearance as seen in the parent strain on SDA plates but with a marked reduction in conidiation, (5.0 × 10^7^ in SG3_col1, 3.2 × 10^7^ in SG3_col2, 1.9 × 10^7^ in SG4_col1, and 4.2 × 10^7^ in SG4_col2) as compared to in the parent strain (2.9 × 10^8^ conidia), after 14 days of growth on SDA plates (Figure 4G). Conidiation was negligible in the mutants on MM agar plates. 

### 3.5. In Vivo Roles: Sensitivity of pacC Mutants to pH Variation and Saline Stress

**Figure 5:**
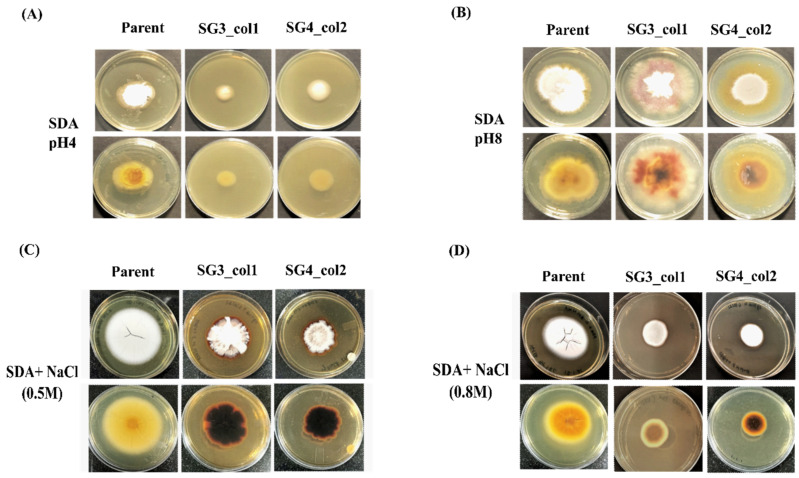
The growth of *pacC* mutants as a function of pH variation and saline stress. The growth of selected *pacC* mutants (SG3_col1 and SG4_col2) was monitored on (**A**) buffered SDA plates at acidic pH (pH 4.0), (**B**) buffered SDA plates at alkaline pH (pH 8.0), (**C**) SDA plates supplemented with 0.5 M NaCl, and (**D**) SDA plates supplemented with 0.8 M NaCl. The top and bottom panels are images of front and reverse sides of the agar plates, respectively. The growth of the parent *T. rubrum* IGIB-SBL-CI1 strain is also shown as a reference control.

In order to monitor the adaptation of mutant strains to pH variation of growth media, the parent strain and the *pacC* mutant strains (SG3_col1 and SG4_col2) were grown on SDA plates at acidic (pH 4) and alkaline (pH8) buffered conditions. At the acidic pH, the growth of mutant strains was severely compromised (mean radial diameter = 17.3 ± 2.1 mm and 24.3 ± 0.6 mm for SG3_col1 and SG4_col2, respectively) in contrast to the parent strain (mean radial growth of 29.7 ± 2.3 mm) (Figure 5A, Figure S5). In addition, the mutants exhibited a smooth surface and absence of pigmentation at the acidic pH as compared to the white-cottony mat with a yellow-brown pigmentation seen in the parent strain (Figure 5A). At alkaline pH, however, more diffused radial growth was observed (Figure 5B, Figure S5) and both the parent and mutant strains retained the respective yellow-brown or dark brown pigmentation as seen during growth on non-buffered SDA plates (Figure 4), suggesting that pacC mutant strains are more sensitive to acidic pH.

The effect of salt stress on SG3_col1 and SG4_col2 mutant strains was similarly evaluated by monitoring the growth on SDA plates (pH 5.6) supplemented with indicated salt concentrations. At 0.5 M NaCl, the mean radial growth of parent strain for three replicates was 56.3 ± 0.6 mm, whereas SG3_col1 and SG4_col2 had mean radial diameters of 45.0 ± 1.7 and 37.3 ± 1.5 mm, respectively (Figure 5C, Figure S5). At both 0.5 and 0.8 M NaCl, an increase in pigmentation was observed, as compared to the parent strain. Further, at 0.8 M NaCl, the mutants exhibited severely stunted growth (mean radial diameters of 25.7 ± 2.5 mm for SG3_col1 and 21.7 ± 1.2 mm for SG4_col2) as compared to 46.3 ± 1.5 mm for the parent strain (Figure 5D, Figure S5), suggesting that *pacC* mutants are sensitive to salt stress as well.

## 4. Discussion

Despite their medical and clinical importance, there exists a lacuna in the molecular analysis of underlying cellular mechanisms among dermatophytes, due to limited methods and poor response to genetic manipulations. In recent years, the CRISPR/Cas9 system has been used for in cellulo genetic manipulations of many organisms due to its versatility and high efficiency. However, the CRISPR/Cas9 system is still in its infancy for large-scale applications in non-model filamentous fungi, viz., dermatophytes. 

In the present work, we used a dual-plasmid-based CRISPR/Cas9 strategy, previously demonstrated in *Aspergillus* species [25,30], for generating mutants of *pacC* in a clinical isolate of *T. rubrum*. The mutagenesis of *pacC* of *T. rubrum* IGIB-SBL-CI1 was brought about by the error-prone NHEJ-dependent repair of double-strand breaks, introduced by separate plasmid-delivered sgRNA and Cas9GFP. While Cas9GFP fusion was expressed from pCas9GFP from a constitutive *PglA* promoter, for monitoring expression by fluorescence microscopy [25], target-specific sgRNA was expressed from a psgRNA5.0 from a highly conserved 5S rRNA promoter. Adaptation of two plasmids in this strategy enables wide applications as one can easily modify the Cas9 protein’s genomic target by simply changing the target sequence present in the sgRNA. The dual-plasmid strategy confers further flexibility in case inefficient sgRNA-guided mutagenesis is observed due to poor expression of Cas9 or sgRNA in the target host cells, as it allows substitution with strong host promoters in the respective vectors, as necessary.

Transformations with linear or circular DNA for bringing about genetic manipulations have often shown variable efficiencies of DNA uptake [41,42]. However, linear plasmids may mimic DNA with a double-strand break, thereby recruiting host repair machinery to this linear-transformed DNA rather than to the target site, resulting in false positives. To overcome the problem of false positives, circular plasmids pCas9GFP and psgRNA5 were used in this study for co-transformation into the protoplasts of *T. rubrum* IGIB-SBL-CI1. In addition, 20 primary transformants of respective sgRNA2-4 were restreaked individually on fresh MMSA plates for two generations (while sgRNA-1 did not express) before detailed characterization. In total, 65–80% of primary transformants were retained suggesting high recovery of mitotically stable mutants. Further screening and selection of generated mutants in most other methods requires extensive sequencing of the obtained transformants. Recently, CRISPR/Cas9-mediated *ura3^−^* auxotrophs were generated to overcome this problem [22]. In the present work described here, an alternate screening method with the help of eGFP, encoded downstream of Cas9, was used. *T. rubrum* IGIB-SBL-CI1 transformants could hence pre-screen positive transformants using detection of GFP fluorescence and overcomes the requirement of prior generation of auxotrophic strains for gene editing (Figure 3A). The T7E1 assay confirmed the *pacC* mutants and indicated 5/16 mutants with sgRNA-3 and 4/13 mutants with sgRNA-4 (Table S4). A frequency of gene disruption of 33.8–37.3% was confirmed using TIDE analysis for both sgRNA-3- and sgRNA-4-mediated mutants (Figure S2). Efficient homologous recombination often requires additional background genetic modifications. For instance, engineering of *ku70* or *ku80* genes in *Arthroderma* spp. or *T. mentagrophytes* significantly increased the homologous recombination frequency in both the dermatophytes to 70% or higher [21,43]. In the absence of any background mutations in *T. rubrum* IGIB-SBL-CI1, the frequency of gene disruption is fairly high and gives the advantage of direct use with clinical strains or for molecular investigations of essential genes and pathways, when complete gene knockout is not possible. 

PacC is required for growth and adaptability of *T. rubrum* on human skin by tailoring its response to environmental pH. During infection, the pathogen breaks down the host proteins on human skin, leading to alkalization, wherein PacC functions by upregulation of alkaline-expressed genes and inhibition of the acid-expressed genes [19,24,40,44]. Earlier disruption of *pacC* of *T. rubrum* resulted in decreased growth on human nails in vitro, decreased conidiation, and decreased secretion of keratinolytic proteases in liquid medium [19]. Mutants of *pacC* by CRISPR/Cas9 editing would hence lead to abnormal PacC translation products; affecting growth, conidiation, hyphal growth, and adaptation at alkaline pH. *pacC* mutants of *T. rubrum* IGIB-SBL-CI1 were a result of a short two-base-pair deletion, generated upstream of PAM in the protospacer region in all the analyzed mutants, as detected using TIDE and ICE web tools. Mutations were generated in exon2 only and revealed a significant decrease in expressed *pacC* transcripts in SG3_col1 and SG4_col2 mutants (Figure 4F). Both *pacC* mutants exhibited reduced growth and several morphological changes in both buffered and non-buffered conditions, as compared to the parent strain, indicating the successful generation of targeted *pacC* mutants in *T. rubrum* IGIB--SBL-CI1 (Figure 4 and Figure 5). The deletion of *pacC* has recently been shown to impair growth under saline stress in a closely related dermatophyte, *T. interdigitale* [40]. In concordance with this, knockout of *pacC* in SG3_col1 and SG4_col2 resulted in severely compromised growth and hyperpigmentation under saline stress as compared to the parent strain (Figure 5C,D). Fungal melanin pigments aid protection from oxidative, UV, and other environmental stress agents in several fungi [45,46]. The observed hyperpigmentation in mutant strains perhaps is a response to mutation stress in these dermatophyte strains. Finally, the absence of any off-target mutations confirmed that the observed phenotypic changes could be ascribed to the CRISPR/Cas9-generated *pacC* mutant colonies (Figure S3). 

## 5. Conclusions

In conclusion, the functional validation of specific gene and gene clusters in non-lab-adapted/clinical isolates of dermatophytes is severely hampered by the lack of efficient genomic tools for making precise genetic modifications. With the advent of CRISPR/Cas9, one can generate site-specific modifications to identify the role of specific genes in a certain pathway and study the molecular mechanisms involved in their function. In this study, a simple and efficient dual-plasmid-based strategy for NHEJ-based gene manipulation of *pacC* in *T. rubrum* IGIB-SBL-CI1 was demonstrated. Mutants were obtained at relatively high frequencies of ~34–37% for gene disruption and showed reduced transcript levels of *pacC* at both acidic and alkaline pH with several morphological abnormalities linked to pH adaptation. As only a few tools are available for large-scale genetic modifications in non-model filamentous fungi, the dual-plasmid-based CRISPR/Cas9 method described here can be used in non-lab-adapted/clinical strains or to examine essential genes when complete gene knockout is not possible. In addition, Cas9GFP avoids laborious screening of mutants, especially when non-template NHEJ repair-based gene editing is employed. We hence propose this method as an alternate tool that can be adapted easily for extensive use for genetic manipulations across *T. rubrum* and related dermatophytes.

## Data Availability

All data generated in this study is contained within the article and available from authors on request.

## Supplementary Materials

The following supporting information can be downloaded at: https://www.mdpi.com/article/10.3390/jof8121241/s1, Figure S1: Schematic of sgRNAs targeting *pacC;* Figure S2: Estimation of indels generated in *pacC;* Figure S3: Off-target analysis; Figure S4: Expression profile of pacC; Figure S5: Radial growth measurements of mutant strains under stress conditions; Table S1: Strains, plasmids, oligonucleotides and primers used in this study; Table S2: List of amplicons and products for in vitro cleavage and T7E1; Table S3: Summary of CRISPR/Cas9-mediated editing of *pacC.*
