# Supplementary material for: A Dual-Plasmid-Based CRISPR/Cas9-Mediated Strategy Enables Targeted Editing of pH Regulatory Gene pacC in a Clinical Isolate of Trichophyton rubrum
Source: J Fungi (Basel). 2022 Nov 24;8(12):1241. doi: 10.3390/jof8121241 (PMC9782554; doi:10.3390/jof8121241)
Supplement: Supplementary file 1 [file jof-08-01241-s001.zip › jof-2018220-supplementary.pdf]

A dual-plasmid-based CRISPR/Cas9-mediated strategy enables targeted editing of pH regulatory gene *pacC* in a clinical isolate of *Trichophyton rubrum*

Supplementary Figures and Tables

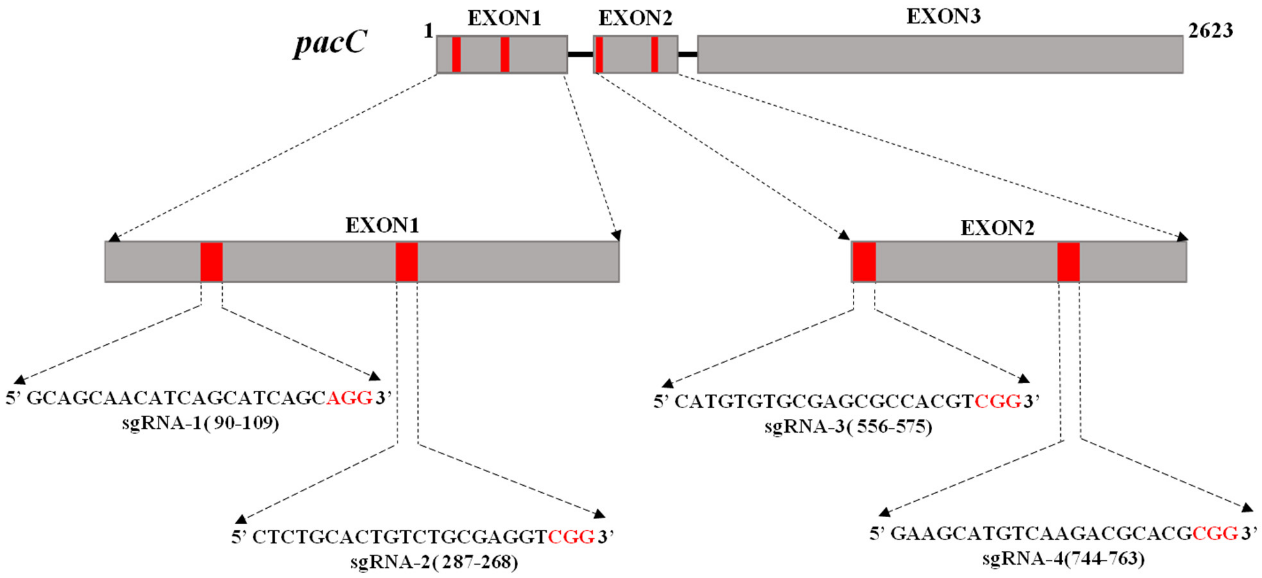

Figure S1: **Schematic of sgRNAs targeting *pacC***. Four sets of oligonucleotides were designed; sgRNA-1 and sgRNA-2 complementary to regions in exon1 and sgRNA-3 and sgRNA-4 complementary to regions in exon2 of *pacC*. Location of the target sites in both exons are marked as red boxes. Sequence of the designed oligonucleotides and their nucleotide positions are indicated with the NGG sequence shown in red.

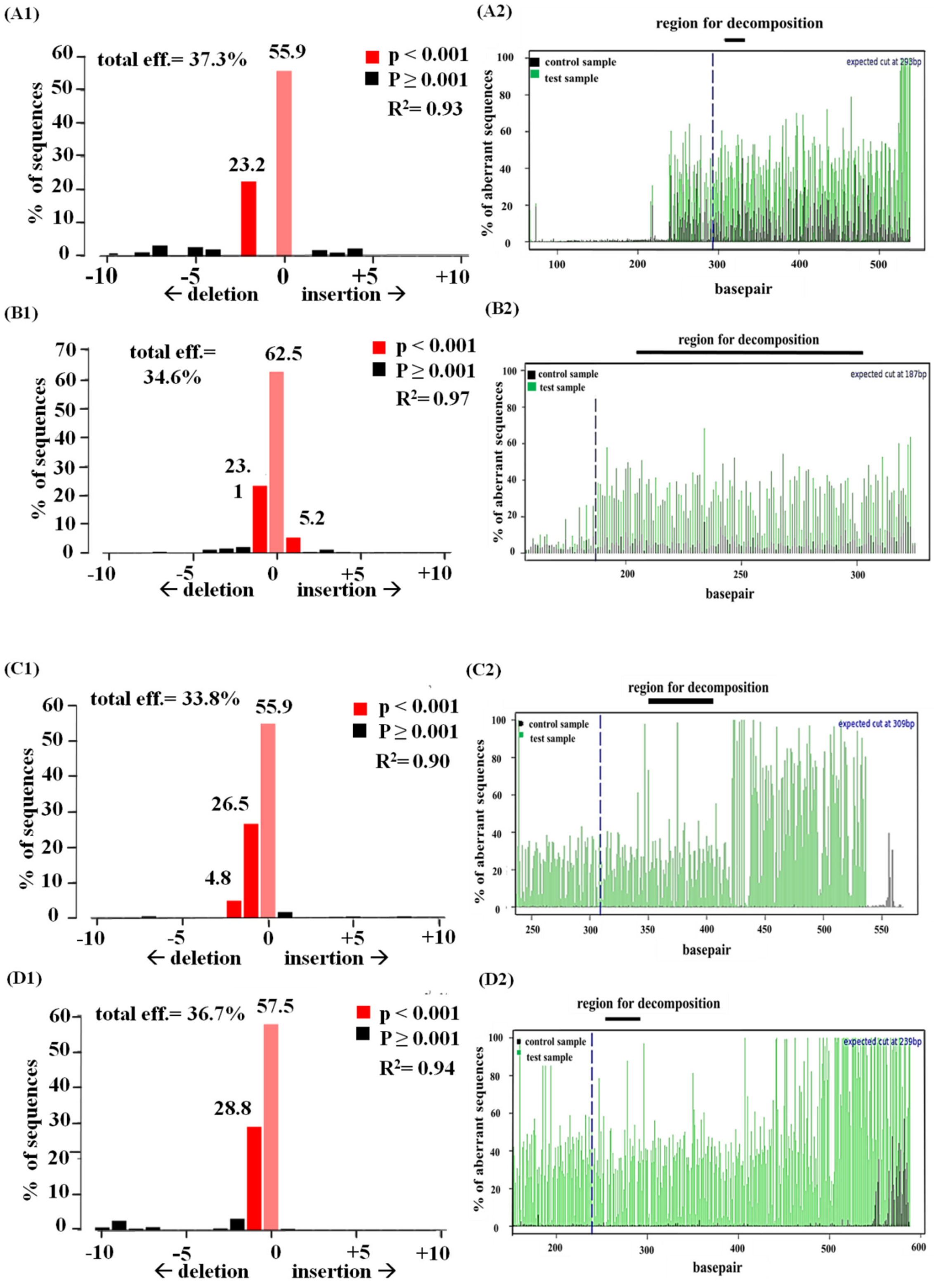

Figure S2: Estimation of indels generated in *pacC*. Indels generated in *pacC* were estimated by TIDE webtool. The estimated mutation efficiency for (A1) SG3_col1, (B1) SG3_col2, (C1) SG4_col1 and (D1) SG4_col2 are indicated in left panels and the respective regions of decomposition are in the accompanying right panels (A2- D2).

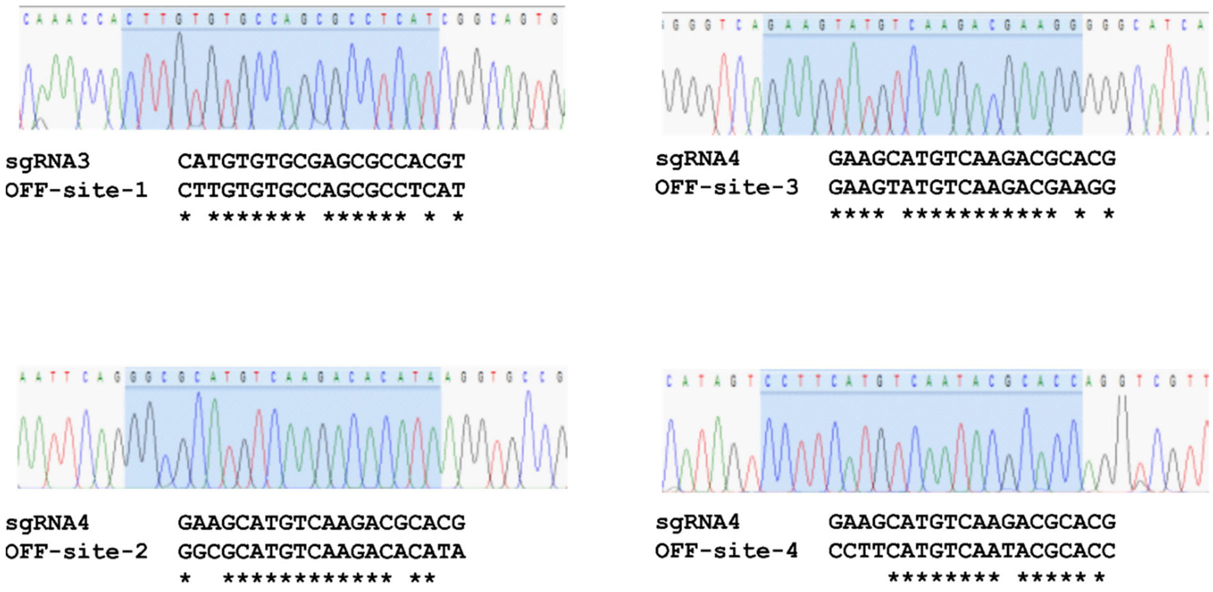

Figure S3: Off-target analysis. Predicted off-target sites were PCR amplified from the mutant colonies, followed by Sanger sequencing of these regions. No mutations were seen in these sites. Sequence alignment of the predicted off-target site with respective sgRNA is also shown in the figure.

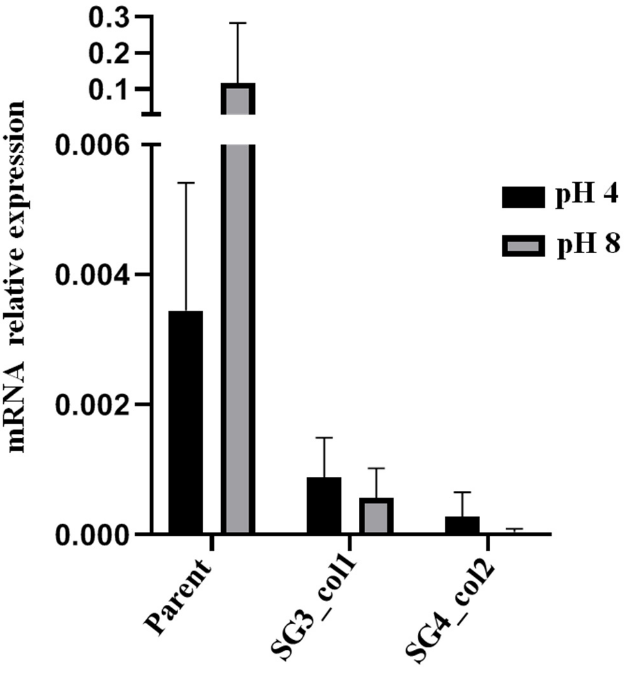

Figure S4: Expression profile of pacC. The expression of pacC indicates expression of pacC in the parent strain is much higher than SG3_col1 and SG4_col2 mutant strains. mRNA expression levels were determined by comparative $2^{- Ct}$ method, normalizing expression to *-actin*. All results represent a mean of a triplicate experiment; error bars indicate standard deviation (±SD) of the triplicate experiments.

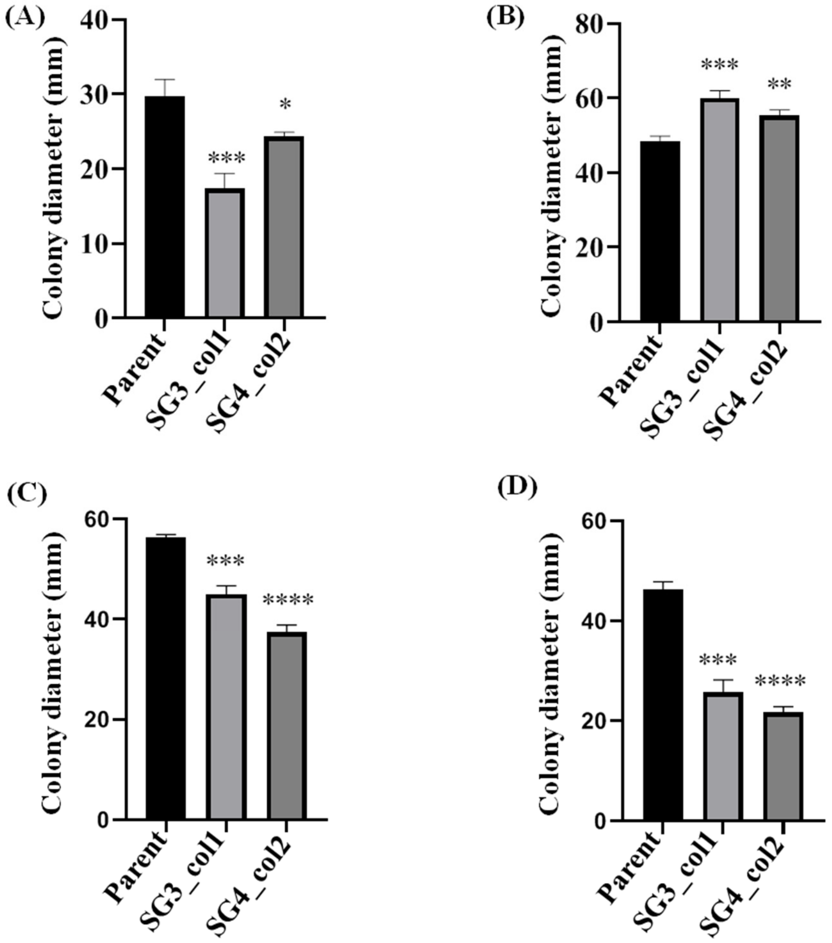

Figure S5: Radial growth measurements of mutant strains under stress conditions. Average colony diameters (in mm) measured from three different plates of each mutant are plotted for growth on (A) buffered SDA plates at acidic pH (B) buffered SDA plates at alkaline pH, (C) SDA plates supplemented with 0.5 M NaCl and (D) SDA plates supplemented with 0.8 M NaCl along with one-way ANOVA analysis was conducted with a post hoc Tukey's test (*$p < 0.05$, **$p < 0.01$, ***$p < 0.001$ and ****$p < 0.0001$).

Table S1: **Strains, plasmids, oligonucleotides and primers used in this study.**

**A. List of Strains**		
**Strain name**	**Description**	**Source**
Escherichia coli DH5*α*	Bacterial strain for maintenance of plasmid constructs (Amp^R)	Laboratory stock
T. rubrum IGIB-SBL-CI1	*T. rubrum* parent strain used in the study (Clinical isolate, maintained at less than three passages in the lab)	Latka et al., 2015

**B. List of Plasmids**		
**Plasmid name**	**Description**	**Source**
psgRNA5.0	Plasmid vector used to clone target-specific sgRNA fragments containing geneticin resistance gene (*neo*^{R})	Zheng et al., 2019
pcas9gfp	Plasmid vector for expression of Cas9 and eGFP fusion (Cas9GFP) *in vivo* with *amdS* marker	Zheng et al., 2018
pT7sgRNA	Plasmid vector for *in vitro* transcription of guide RNA products in order to carry out *in vitro* cleavage assay (*Amp*^{R})	(Jao et al., 2013)

**C. List of Primers and oligonucleotides used in the study**		
**Primer name**	**Description**	**Sequence**
sgRNA1FP	Primer for cloning sgRNA-1 in psgRNA5.0 and pT7sgRNA	CACCGCAGCAACATCAGCATCAGC
sgRNA1RP	Primer for cloning sgRNA-1 in psgRNA5.0 and pT7sgRNA	AAACGCTGATGCTGATGTTGCTGC
sgRNA2FP	Primer for cloning sgRNA-2 in psgRNA5.0 and pT7sgRNA	CACCGTCTGCACTGTCTGCGAGGT

sgRNA2RP	Primer for cloning sgRNA-2 in psgRNA5.0 and pT7sgRNA	AAACACCTCGCAGACAGTGCAGAG
sgRNA3FP	Primer for cloning sgRNA-3 in psgRNA5.0 and pT7sgRNA	CACCGATGTGTGCGAGCGCCACGT
sgRNA3RP	Primer for cloning sgRNA-3 in psgRNA5.0 and pT7sgRNA	AAACACGTGGCGCTCGCACACATG
sgRNA4FP	Primer for cloning sgRNA-4 in psgRNA5.0 and pT7sgRNA	CACCGAAGCATGTCAAGACGCACG
sgRNA4RP	Primer for cloning sgRNA-4 in psgRNA5.0 and pT7sgRNA	AAACCGTGCGTCTTGACATGCTTC
PSGRNA5FP	Forward primer for sequencing sgRNAs cloned in psgRNA5.0	GGTTGGAGATTCCAGACTCAGC
PSGRNA5RP	Reverse primer for sequencing sgRNAs cloned in psgRNA5.0	AAGCACCGACTCGGTGCC
PT7sgRNAFP	Forward primer for sequencing sgRNAs cloned in pT7sgRNA	GGCCGATTCATTAATGCAGCT
PT7sgRNARP	Reverse primer for sequencing sgRNAs cloned in pT7sgRNA	ACTGAGAGTGCACCATATGC
G1FP	Forward primer for respective amplicons for *in vitro* cleavage and T7E1 assay	TCTATCAGCGCTGCCTGTCT
G1RP	Reverse primer for respective amplicons for *in vitro* cleavage and T7E1 assay	CCACTGACATTCGAGCGAG
G3RP	Reverse primer for respective amplicons for *in vitro* cleavage and T7E1 assay	TGAGCGGCTATAACGTTGTC

G4FP	Forward primer for respective amplicons for *in vitro* cleavage and T7E1 assay	GCTCGCTCGAATGTCAGTG
G4RP	Reverse primer for respective amplicons for *in vitro* cleavage and T7E1 assay	GAGGCTGGTAATACTGTGGTG
ActinRTFP	Forward primer for quantitative reverse transcriptase PCR of *-actin*	GAATCTCCCCCCTCATGCTT
ActinRTRP	Reverse primer for quantitative reverse transcriptase PCR of *-actin*	GCCTGGGCTTCCGACACT
PacCRTFP	Forward primer for quantitative reverse transcriptase PCR of *pacC*	TCCCAGCAGCCCCAAC
PacCRTRP	Reverse primer for quantitative reverse transcriptase PCR of *pacC*	ATGTGGGAGGTGATGTGGT

(Nucleotides highlighted in yellow depicts overhangs for Bbs1 enzyme enabling cloning of these oligonucleotides in expression vectors used in this study).

Table S2. List of amplicons and products for *in vitro* cleavage and T7E1.

**PCR Amplicons**	**Region of *pacC* amplified**	**Primers used for in vitro cleavage/ T7E1 assay**	**Amplicon size**	**Size of cleaved fragments**
G1	-319 to 286	G1 FP + G1RP	727	425 + 302
G2	-319 to 408	G1 FP + sgRNA3RP	894	589 + 305
G3	267 to 945	sgRNA2FP + G3RP	679	306 + 373
G4	389 to 1016	G4FP + G4RP	628	372 + 256

Table S3. Summary of CRISPR/Cas9-mediated editing of *pacC*.

Plasmid name	Protospacer and PAM sequence [a]	Number of analyzed transformants	Mitotically-stable transformants	Mutants detected by T7E1	Mutations screened by Sequencing
psgRNA5_sg1	GCAGCAACATCAGCATCAGCAGG	0	0	0	0
psgRNA5_sg2	CTCTGCACTGTCTGCGAGGTCGG	20	16	0	0
psgRNA5_sg3	CATGTGTGCGAGCGCCACGTCGG	20	16	5	5 [b] (2 bp del)
psgRNA5_sg4	GAAGCATGTCAAGACGCACGCGG	20	13	4	4 [c] (2 bp del)

a- PAM sequence (NGG) is shown in red. b- Sequencing identified a 2bp deletion in all the screened colonies. c - Sequencing identified a 2bp deletion in all the screened colonies.
